# Translating Virtual Prey-Predator Interaction to Real-World Robotic Environments: Enabling Multimodal Sensing and Evolutionary Dynamics

**DOI:** 10.3390/biomimetics8080580

**Published:** 2023-12-01

**Authors:** Xuelong Sun, Cheng Hu, Tian Liu, Shigang Yue, Jigen Peng, Qinbing Fu

**Affiliations:** 1 Machine Life and Intelligence Research Center, Guangzhou University, Guangzhou 510006, China; xsun@gzhu.edu.cn (X.S.); c_hu@gzhu.edu.cn (C.H.); sy237@leicester.ac.uk (S.Y.); 2 School of Mathematics and Information Science, Guangzhou University, Guangzhou 510006, China; 3 School of Mechanical and Electrical Engineering, Guangzhou University, Guangzhou 510006, China; 4 MLTOR Numerical Control Technology Co., Ltd., Zhongshan 528400, China; liutian@mail.mltor.com; 5 School of Computing and Mathematical Sciences, University of Leicester, Leicester LE1 7RH, UK

**Keywords:** prey-predator interaction, agent-based approach, swarm robotics, multi-modal interaction, emergent behavior, bio-robotics, artificial life, adaptive behavior

## Abstract

Prey-predator interactions play a pivotal role in elucidating the evolution and adaptation of various organism’s traits. Numerous approaches have been employed to study the dynamics of prey-predator interaction systems, with agent-based methodologies gaining popularity. However, existing agent-based models are limited in their ability to handle multi-modal interactions, which are believed to be crucial for understanding living organisms. Conversely, prevailing prey-predator integration studies often rely on mathematical models and computer simulations, neglecting real-world constraints and noise. These elusive attributes, challenging to model, can lead to emergent behaviors and embodied intelligence. To bridge these gaps, our study designs and implements a prey-predator interaction scenario that incorporates visual and olfactory sensory cues not only in computer simulations but also in a real multi-robot system. Observed emergent spatial-temporal dynamics demonstrate successful transitioning of investigating prey-predator interactions from virtual simulations to the tangible world. It highlights the potential of multi-robotics approaches for studying prey-predator interactions and lays the groundwork for future investigations involving multi-modal sensory processing while considering real-world constraints.

## 1. Introduction

Prey-predator interaction have long captivated researchers due to their crucial role in driving organism’s evolution and shaping functional ecosystems [1,2,3,4]. Previous studies investigating predator-prey dynamics have utilized various approaches, including observational studies [5], experimental manipulations [6,7,8], and mathematical modeling [9,10,11,12,13], with recent advancements in agent-based simulations using virtual agents [14,15] or real robots [16]. While previous studies have examined population dynamics in prey-predator interactions using mathematical models, there has been limited exploration of multi-modal interactions within populations. Animals rely on multiple sensory signals for survival, particularly vision [17] and olfactory [18,19]. In some cases, vision and olfactory work in tandem to aid decision-making [20,21]. However, implementing interaction mechanisms involving multi-modal sensory interaction is challenging due to its complexity and the difficulties of being accurate. Therefore, almost no research has been conducted on this, resulting in the vagueness of its characteristics in prey-predator interaction systems.

The recent development of agent-based simulation [22,23] and the robotics technology [24,25,26,27,28,29] provides an opportunity to devise a system that could conduct real-time and real-world experiments of prey-predator interaction scenarios with multi-modal sensory and evolutionary processing involved. We highlight that bringing the multi-modal prey-predator interaction studies from virtual computer to real physical world matters at least in the following aspects: (1) Robots, designed to navigate complex environments, offer a more ***precise depiction*** of interactions than any other simulations [30,31,32,33]. (2) real-world interactions with physical constrains often lead to ***emergent behaviors*** [34,35]; (3) Robots can embody various predator and prey types, enabling exploration of how diversity shapes interactions through adaptable sensory processing and control mechanisms, exemplifying the ***embodiment intelligence*** [29,36,37]; (4) Robots equipped with sensors, replicate the sensory capabilities of real organisms, such as detecting prey movement or tracing scent trails; (5) Robotics approach fosters collaboration among biologists, engineers, and computer scientists. This ***multidisciplinary approach*** may yield innovative solutions and deeper insights into interactions.

To harness the full potential of robotics technology for investigating multi-modal prey-predator interactions, this paper employs a swarm robotics platform to conduct targeted experiments. Our focus is on validating the capability of the proposed system to facilitate multi-modal and evolutionary prey-predator studies. To achieve this, we devised a dedicated prey-predator interaction system wherein prey possess evolutionary attributes and the ability to sense both visual and olfactory cues in their environment. This tailored system was implemented both in computer simulations and the real physical realm.

We initially employed computer simulations to systematically explore the influence of environmental factors and predator hunting traits on prey dynamics and the overall prey-predator interaction system. Subsequently, to affirm the viability of swarm robotics in studying prey-predator interactions, we replicated select simulation outcomes using mobile robots in real-world scenarios. The findings, encompassing emergent behaviors, consistent preferences, and identifiable patterns in response to specific environmental factors, validate the effectiveness of our proposed robotics system in enabling multi-modal and evolutionary prey-predator interaction studies.

In summary, this paper introduces a specialized multi-agent robotics system designed for investigating prey-predator interactions. This approach integrates multi-modal sensory input and evolutionary processing, effectively bridging the gap between computer simulations and real-world prey-predator dynamics. The results demonstrate the potential of this methodology to provide a valuable complement to traditional approaches, enhancing the study of complex dynamics within prey-predator systems.

## 2. The Devised Prey-Predator Interaction with Multi-Modal and Evolutionary Processing

This section establishes fundamental agent and world definitions, outlining the interactions between prey and predator agents. The introduced metrics for evaluating the dynamic system’s performance are also presented. For more comprehensive details and mathematical descriptions, refer to Appendix A.

### 2.1. Computer Simulation

**Figure 1:**
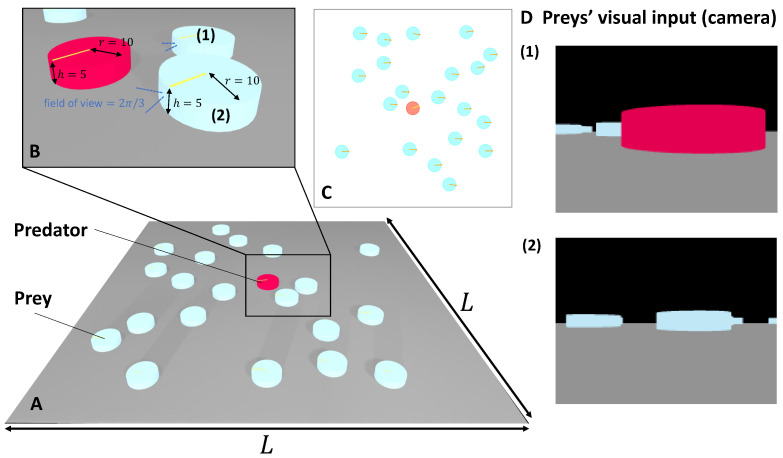
Agent Definition and Simulated 3D World: (**A**) Depiction of the simulated 3D world featuring designated agents. (**B**) Close-up perspective of the area outlined in (**A**), illustrating the geometric attributes of preys and predators, along with the visual field of view of the prey’s camera (indicated by dashed blue lines). (**C**) Overhead view of the simulated 3D world and its agents, as presented in (**A**). (**D**) Instances of vision input from preys positioned and oriented as demonstrated in (**B**), annotated as (**1**) and (**2**) correspondingly. The image dimensions are 200×150.

The agent population consists of two categories: prey and predator. Preys are represented as cyan cylinders with a radius of 10 and height of 5, while predators are denoted by red cylinders of the same dimensions (as depicted in Figure 1). A yellow bar positioned atop the cylinder signifies the agent’s current heading direction. Notably, the simulation does not incorporate any physical constraints, allowing agents to potentially collide without exerting forces upon each other. Consequently, an agent’s motion is entirely dictated by the control strategy implemented.

**Figure 2:**
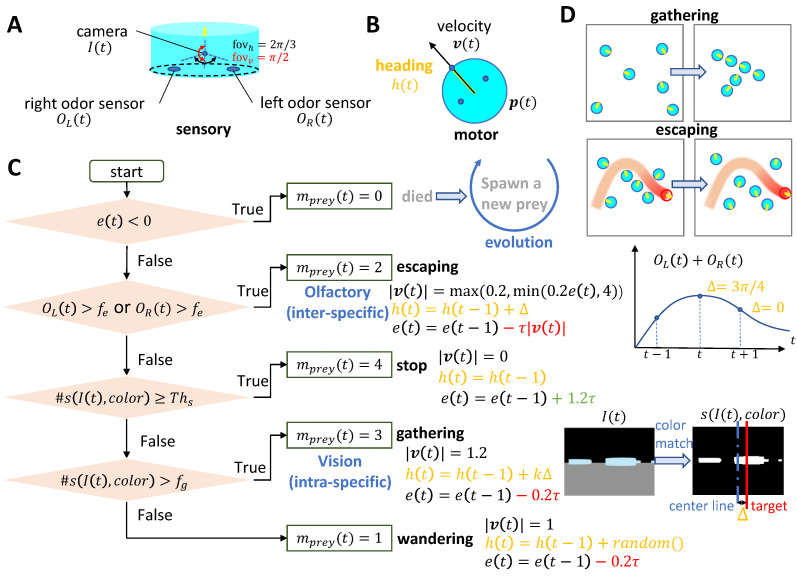
Sensory-motor loop and delineated behaviors of the **prey**: (**A**) Each prey individual possesses a camera (visual input) featuring a horizontal field of view of 120 degrees and a vertical field of view of 90 degrees. Additionally, two odor sensors (olfactory input) are symmetrically placed at the agent’s base. (**B**) Motion attributes defined within the prey agent. (**C**) Flowchart depicting the determination of motion state $m_{prey}\left(t\right)$, the update rule of energy e(t), and the corresponding motion control: velocity v(t) and heading direction h(t). I(t) denotes the present visual input, while s(I(t),color) refers to a binary image following color matching. #(x) represents the proportion of non-zero pixels to the total pixels (i.e., the image size of 200×150). (**D**) Representative spatial distribution of the prey population, influenced by visual aggregation (**upper panel**) and olfactory escape (**lower panel**). Orange text indicates the heading direction control, blue text describes state-related control, red and green text represent energy consuming and obtaining respectively.

Each member of the prey population is equipped with multi-modal sensory inputs—visual and olfactory (as depicted in Figure 2A)—that dictate their motion behavior. Specifically, visual cues steer task-driven aggregation, while olfactory cues, emitted by predators, trigger escape responses (as shown in Figure 2C,D). In instances where sensory inputs lack sufficient strength, preys adopt a wandering behavior within the arena, changing their heading direction randomly at each time step. The highest priority is accorded to the escape behavior (when $m_{prey}\left(t\right)=2$). This signifies that once the prey’s perception of odor strength surpasses the threshold ($f_{e}$, also known as the escaping preference factor), the escape behavior is activated. The agent then abruptly alters its heading if the temporal change in the combined value of the left and right odor sensors is positive. Otherwise, the agent retains its previous course. Conversely, vision-based intra-specific gathering behavior ensues in the absence of detected odor but in the presence of a visually recognized color (associated with the body color of mates) within its visual field. Activation of this behavior is determined by a threshold ($f_{g}$, or gathering preference factor), representing the percentage of pixels with the chosen color relative to the entire visual image. Gathering movements prompt preys to converge on their mates, and the agent halts its movement when a target mate appears sufficiently large in their field of view (dictated by the threshold $Th_{s}$). Energy generation and consumption are intrinsically tied to the prey’s motion state. To introduce selective pressure against the escape behavior and stimulate evolutionary processes, energy consumption is proportionate to the escape speed. For other instances (wandering and gathering), energy consumption remains constant (0.2τ). Following cessation of movement, the prey’s energy is replenished at a rate of 1.2τ (refer to red and green text in Figure 2C for energy consumption and generation details, respectively). This framework introduces a trade-off: gathering behavior replenishes energy while evading predators consumes it. The resulting conflict in selective pressures may guide the evolution of distinct behavioral directions.

**Figure 3:**
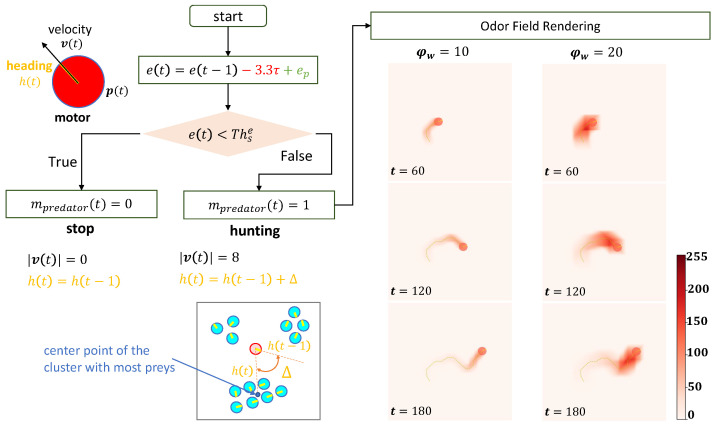
Motor (velocity v(t) and heading h(t)), energy e(t) and defined behaviors of the **predator**, along with instances of established odor fields exhibiting different odor injection widths ($\phi_{w}=10$ and $\phi_{w}=20$; Equation (A7)) but sharing the same motion trajectory. The predator’s motor behavior is akin to that of the prey. The predator is denoted by a red circle with a yellow bar, while cyan circles represent preys. Odor fields are visualized as heatmaps, with the predator’s current position and historical trajectories superimposed. Orange text indicates the heading direction control, red and green text represent energy consuming and obtaining respectively.

The predator’s behavior outlined in this study is relatively straightforward compared to that of the prey, mainly due to the absence of egocentric sensory information. Predators move freely within the arena, aiming to capture preys, unless their energy surpasses the threshold $Th_{s}^{e}$. During hunting, the predator’s motion direction aligns with the vector pointing from its present position p(t) to the geometrical center of the cluster containing the largest number of preys (represented by the blue point marker in Figure 3). While cluster and prey positions are manually provided in this study, in realistic contexts, the predator could obtain such information via its own sensory system. Energy expenditure in the predator is linked to odor emission (at a constant rate of 3.3τ) and is replenished through digestion of preys within the range of its odor field ($e_{p}$ in Figure 3, for detailed information, refer to Appendix A.2.1).

### 2.2. Real Robot Implementation

**Figure 4:**
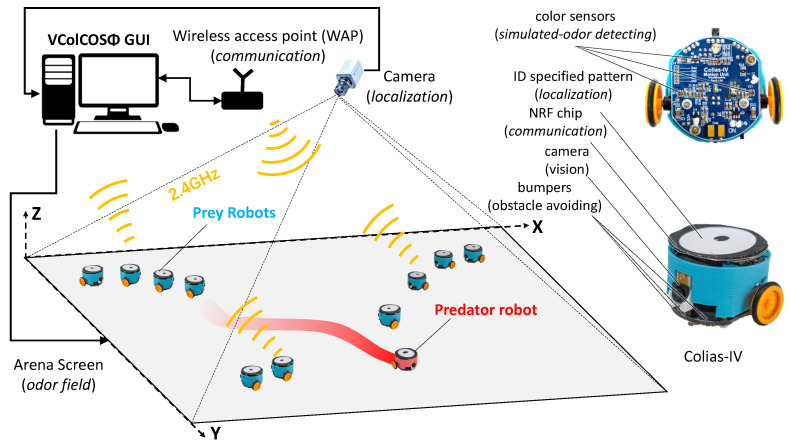
The swarm robotics platform VColCOSΦ platform [25,26] employed for conducting the prey-predator interaction scenario defined in this study.

To realize the prey-predator interaction scenario delineated in this study within the physical realm, a robotics platform equipped with odor field rendering, localization, and visual sensing capabilities is essential. Among available swarm robotics platforms, the recently developed VColCOSΦ platform [25,26] aligns better with these requirements. This platform was meticulously designed for conducting swarm robotics research, equipped with the capability to construct and detect multi-modal information. This includes rendering optic-simulated pheromone/odor fields and visual scenes. The mobile robot involved, referred to as Colias-IV [28], is adept at sensing optic-simulated odors through color sensors and capturing visual cues with its embedded camera. As demonstrated in Figure 4, the predator robot, adorned with a red shell, can be dynamically localized to render the odor field, providing the sensory stimuli required for preys’ *olfactory escaping* task. Prey robots, encased in cyan shells, serve as color targets for their *visual gathering* task.

**Figure 5:**
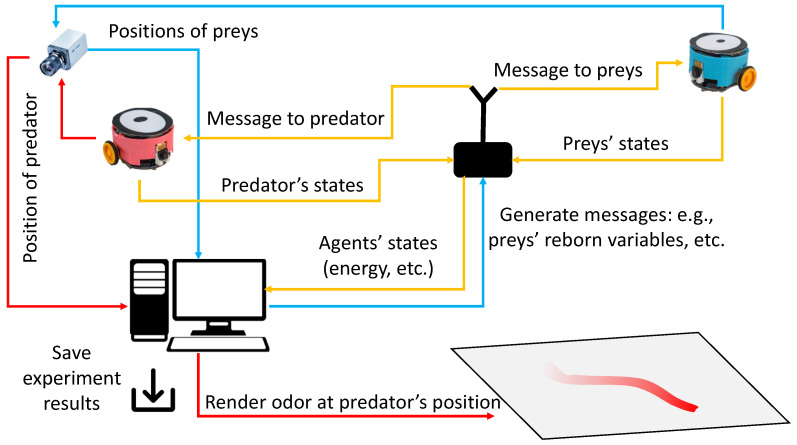
The schematic diagram of the information flow in the proposed robot experiment implementing the defined prey-predator interaction scenario.

**Figure 6:**
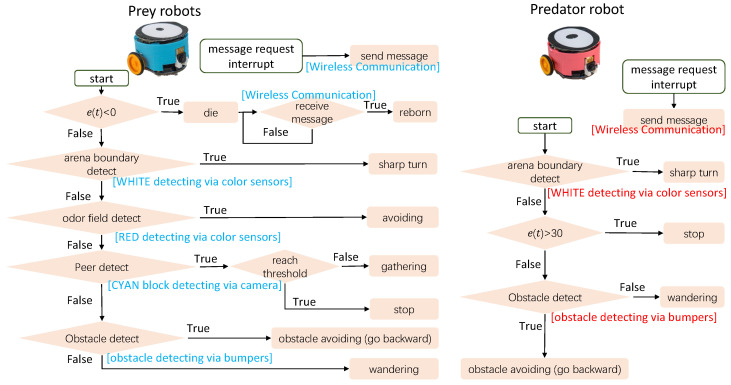
The flowcharts of the control strategy of prey (left) and predator (right) robots. The applied hardware modules are labeled with text in brackets (cyan text for preys and red text for predator) alongside the corresponding function required in the control algorithm. e(t) refers to the energy value of the agent.

The host computer, where the VColCOSΦ GUI operates, facilitates message exchange with all robots via the wireless communication channel, enabling real-time command transmission and inner state monitoring. This capability facilitates the efficient execution of complex experiments involving multiple robots. As exemplified in this study, the information flow within the system is illustrated in Figure 5, which can be distilled into four key components: (1) **localization system** precisely determines the real-time positions of the robots; (2) odor fields are dynamically rendered based on the predator’s current location via the **odor rendering system**; (3) **two-way communication system** enables the host computer to both monitor the agents’ states and convey real-time data and commands; (4) **mobile robots** endowed with visual and optic-simulated odor sensing capabilities, fulfill the roles of both prey and predators in the designed experiments. For an in-depth understanding of the control strategies employed by both prey and predator robots, see Figure 6.

Note that there are several distinctions exist between the computer simulation and the real robot experiments: (1) In the computer simulation, predators can track prey clusters. However, in real robot experiments, transmitting position information via wireless communication incurs time delays that hinder robust control algorithms. Consequently, real robot predators behave by running randomly within the arena (see Figure 6); (2) In real robot experiments, the death of a prey robot is simulated, involving a message with evolved behavioral factors (gathering and escaping preference factors) sent from the host computer to the “dead” prey robot. The robot is then reborn and resumes its designated task (see Figure 6); (3) In real robot experiments, arena boundaries are indicated by white bordered rectangles, detected by robots’ color sensors to trigger sharp turns and avoid collisions; (4) Due to bandwidth and communication delay, real robot experiments involve limited robot numbers and potentially delayed data on the host computer, compared to the simulation; (5) Real robot cameras have a resolution of 99×72 and a horizontal field of view of $70^{\circ }$, differing from the 200×300 resolution and $120^{\circ }$ field of view in simulations; (6) In contrast to simulations, real robot collisions occur. Bumpers are added to mitigate this, although the peripheral wheel placement on the Colias robots occasionally results in entanglements; (7) The message exchange interval between robots and host computer in real experiments is 1 s, considerably lower than the simulation’s 30 ms. However, robot sensor updates and motion control remain at the millisecond level.

## 3. Results of Computer Simulation

**Figure 7:**
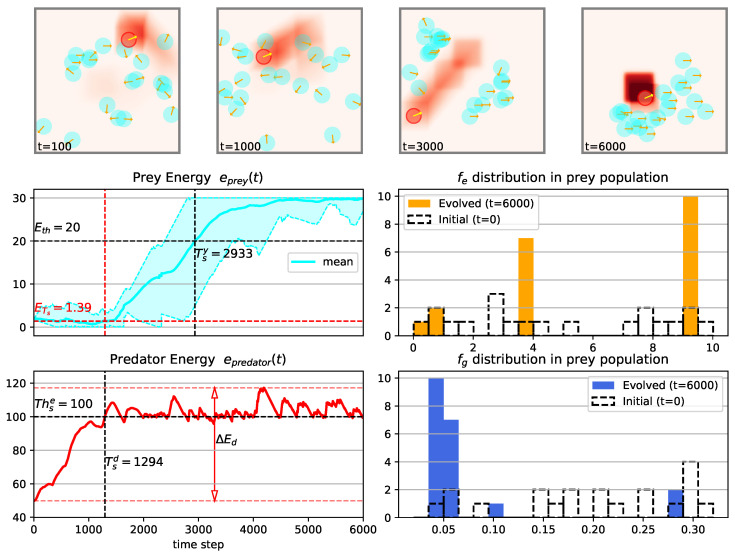
The results of one example simulation using parameters in Table A1 with arena length L=200 and odor injection width $\Phi_{w}=20$. The upper four subplots provide a bird-eye view of the simulated 3D world, showcasing the positions and headings (orange arrows) of preys and predators, as well as the odor field at labeled time-steps. The lower left panel displays the energy profiles of each population (cyan curves for prey population’s maximum, mean, and minimal energy; red curves for predators with dashed red lines depict the maximum and minimum value of predator’s energy) over time, with relevant metrics annotated. The lower right panel illustrates the initial (black-dotted bars) and final (bars) distributions of evolving traits (upper for escaping preference factor $f_{e}$ and lower for gathering preference factor $f_{g}$) within the prey population.

**Figure 8:**
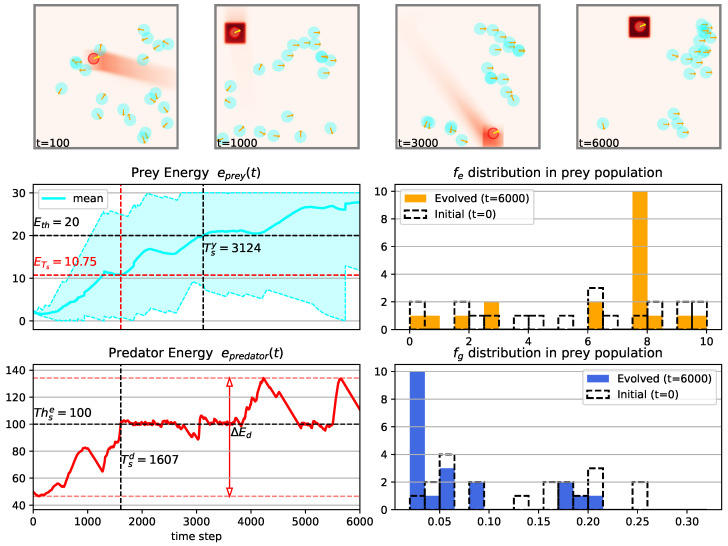
The results of one example simulation using parameters in Table A1 with arena length L=280 and odor injection width $\Phi_{w}=20$. Meanings of sub-figures are the same as that of Figure 7. But note that in the upper four plots, the size of the arena and agent’s bodies are scaled.

Figure 7 presents an overview of the outcomes from a simulation conducted using the specified agents, environment, and parameters listed in Table A1, with an arena length of L=200 and odor injection width $\Phi_{w}=20$. For an animated example of the simulation process, refer to Video S1 in the supplementary materials. Results of another simulation conducted in a larger arena (L=280) is shown in Figure 8. $T_{s}^{y}$, $T_{s}^{d}$, $E_{T_{s}}$ and $\Delta E_{d}$ are defined metrics to evaluate the dynamics of population energy and they are formulated as follows:

(1) The initial time step when the energy of prey and predator attains $E_{th}=20$ and $Th_{s}^{e}=100$ respectively,

(1) $$\begin{array}{c} T_{s}^{y}=inf\left\{t|e_{prey}\left(t\right)\geq E_{th}\right\}\\ T_{s}^{d}=inf\left\{t|e_{predator}\left(t\right)\geq Th_{s}^{e}\right\} \end{array}$$

(2) The average energy of the prey population at time step $T_{s}^{d}$:

(2) $$E_{T_{s}}=e_{prey}\left(T_{s}^{d}\right)$$

(3) The difference between the lowest and highest energy of the predator during the whole simulation:

(3) $$\Delta E_{d}=\max e_{predator}\left(t\right)-\min e_{predator}\left(t\right)$$

The distribution of the escaping and gathering preference factors of the prey agents is presented through histogram plots in the lower right panel of both Figure 7 and Figure 8. We divided the data into 20 bins and the bin width for escaping and gathering factor is 10/20=0.5 and 0.3/20=0.015 respectively.

The initial values of the escaping and gathering preference factors are sampled from a uniform distribution, but they converge after the evolution process. This convergence highlights the effectiveness of the evolutionary mechanism driven by the selective pressure imposed by the prey-predator interactions. It is evident that the preys exhibit a tendency to gather around the odor field released by the predator, giving rise to distinct spatial patterns formed through the prey-predator interactions (as observed in the upper four subplots of Figure 7 and Figure 8). The temporal evolution of both the prey population’s energy and the predator’s energy also reveals intriguing tendencies that merit further investigation. For instance, a comparison between the simulation results with an arena size of L=280 (Figure 8) and a smaller arena size of L=200 demonstrates that the mean energy of the prey population ${\bar{e}}_{prey}\left(t\right)$ at the time when the predator’s energy $e_{predator}\left(t\right)$ reaches 100 is lower in the smaller arena. This observation suggests that a smaller arena, which imposes stronger environmental constraints, hampers the development of the prey population. However, it’s noteworthy that the time it takes for the prey population to surpass the energy threshold (${\bar{e}}_{prey}\left(t\right)\geq 20$) is earlier in the smaller arena. This discrepancy might be attributed to the predator’s behavior, as the predator’s energy exceeds its stopping threshold ($Th_{s}^{e}$) sooner in the smaller arena.

### 3.1. Temporal Dynamics of Population Energy

**Figure 9:**
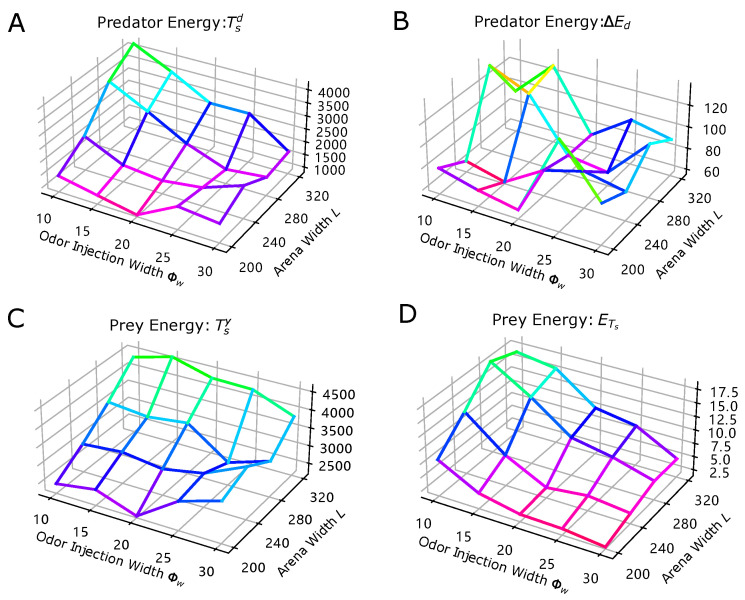
The results of the systematic investigation for evaluating the environmental factor (arena length *L* in *x*-axis) and the predator’s odor injection width ($\Phi_{w}$ in the *y*-axis)’s effect to the temporal dynamics of the prey-predator interaction using the defined metrics. Vertices in the plots are determined by the averaged values (lighter green colors represent higher values while darker red colors indicate lower values) of 10 trials for every parameters setting of the simulation. (**A**–**D**) corresponds to metrics formulated in (1)–(3).

To further investigate the dynamics of population energy observed in the simulations, we conducted systematical experiments by varying the arena length (*L*) and the odor injection width ($\Phi_{w}$) of the predator. Results are shown in Figure 9. Specifically, the temporal behaviors of prey and predator’s energy accumulation at certain time steps ($T_{s}^{y}$ in Figure 9C and $T_{s}^{d}$ in Figure 9A) exhibit noticeable trends as these parameters are varied. Generally, as the arena length increases, the time steps required for both populations to attain specific energy levels tend to increase as well. However, the relationship between the odor injection width and these time steps is more intricate, revealing local minima when the arena length (*L*) is 280 and the odor injection width ($\Phi_{w}$) is 25. On the other hand, the prey population’s average energy ($E_{t_{s}}$ in Figure 9D) at the time when the predator’s energy reaches the threshold ($Th_{s}=100$) exhibits a more straightforward trend. It tends to decrease with larger odor injection widths and smaller arena lengths. Nevertheless, the disparity between the predator’s maximum and minimum energy values ($\Delta E_{d}$ in Figure 9B) throughout the simulation does not exhibit a clear trend. This suggests that this variable may be influenced by other parameters, such as the motion speed of both predators and preys (|v(t)|), and may require further investigation.

### 3.2. Evolution Process in Prey Population

To investigate the effect of environmental factor and the predator’s trait to the prey evolution process, we first apply the histogram calculation, then the maximum number of $N^{j}$ at the initial (t=0) and final (t=6000) time step is computed:

(4) $$\begin{array}{c} M\left(f_{e},t\right)=\max N_{f_{e}}^{j}\left(t\right)\\ M\left(f_{g},t\right)=\max N_{f_{g}}^{j}\left(t\right) \end{array}$$

then we get the difference:

(5) $$\begin{array}{c} \Delta M\left(f_{g}\right)=M\left(f_{g},6000\right)-M\left(f_{g},0\right)\\ \Delta M\left(f_{e}\right)=M\left(f_{e},6000\right)-M\left(f_{e},0\right) \end{array}$$

to quantify the extent of change (evolution). A higher value indicates a greater degree of evolution, implying that more prey agents’ $f_{g}$ and $f_{e}$ values are converging to the same range.

**Figure 10:**
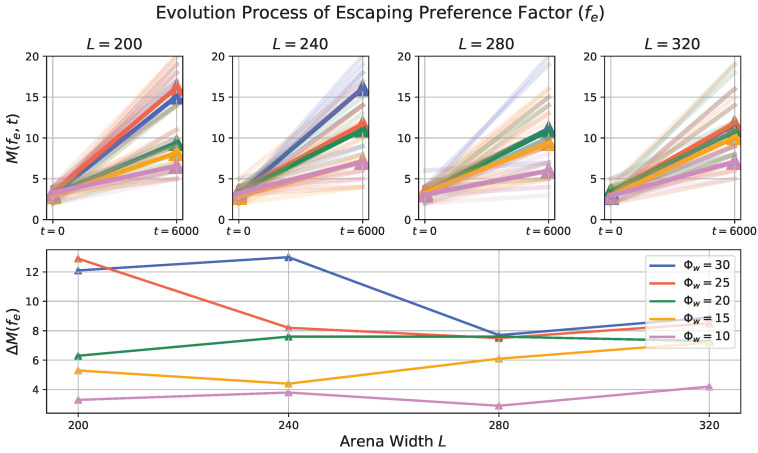
Results of the preference factor in the evolution process. The upper four subplots illustrate the maximum number of agents within each bin of the histogram calculation (refer to (4)) at the initial (t=0) and final (t=6000) time steps. The simulations are conducted with varying arena lengths (L=200,240,280,320 from left to right) and different odor injection widths, each encoded by a specific line color. Each line corresponds to one simulation trial, with the highlighted line indicating the mean value of 10 trials. The lower subplot of Figure 10 depicts the metric representing the difference between the maximum number of agents in each bin at the initial and final time steps (calculated according to (5)). This analysis offers insights into how the combination of arena length and odor injection width impacts the evolution of the escaping preference factor in the prey population.

**Figure 11:**
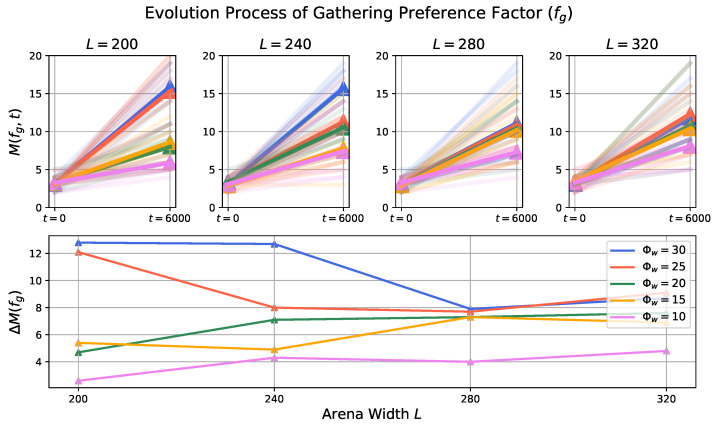
The arena length and predator’s odor injection width that act as the selective pressure to the prey population have effects on their degree of evolution process for gathering preference factor. Description of the figure is same as that of Figure 10.

Figure 10 and Figure 11 visually represent the influence of external factors on the evolutionary processes of the prey population’s behavioral traits—escaping and gathering preference factors, respectively. These outcomes indicate that, in general, larger odor injection widths coupled with smaller arena lengths tend to exert higher selective pressure, thereby resulting in a more pronounced degree of evolution. Notably, it appears that the impact of odor injection width as a dynamic external factor on the prey population’s evolutionary degree is more potent compared to that of the arena length. Nevertheless, the relationships between these two factors and the extent of evolution do not follow linear and monotonic patterns, suggesting the existence of intricate inherent characteristics within the established prey-predator interaction.

The evolution degree ΔM of both the escaping preference factor ($f_{e}$) and the gathering preference factor ($f_{g}$) exhibits a similar trend as changes occur in the arena length and odor injection width. This observation implies that genotypes that respond to relevant or analogous environmental factors may evolve concurrently to achieve an optimal fitness. In other words, as the environmental conditions, represented by the arena length and odor injection width, vary, the behavioral traits of the prey population adapt and evolve in a coordinated manner to better suit the prevailing conditions and enhance their overall fitness.

**Figure 12:**
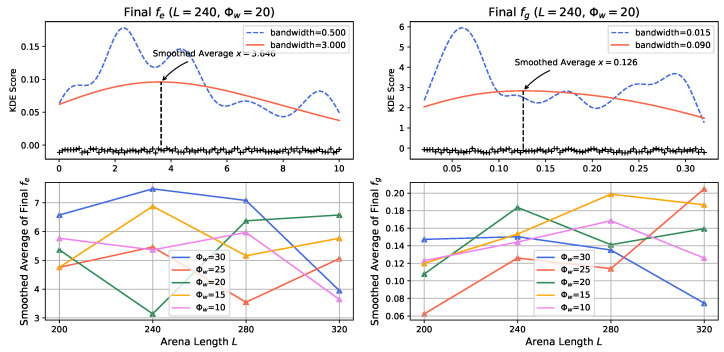
The Kernel Density Estimation (KDE) analysis of the evolved escaping (**left panel**) and gathering (**right panel**) preference factor. Upper plots illustrates how to get the smoothed average using higher bandwidth exemplified by simulation with arena length L=240 and odor injection factor $\Phi_{w}=20$. Cross makers depict the individual value of $f_{e}$ and $f_{g}$ in the prey population.

To gain further insights into the evolved genotype of the prey population under varied external factors, we utilize Kernel Density Estimation (KDE) on the final values of alive prey agents’ escaping and gathering preference factors. We apply a Gaussian kernel with a bandwidth six times higher than the standard bandwidth to obtain a smoothed average value of $f_{e}$ and $f_{g}$ at the final time step of simulations. The results of simulations with different arena lengths and odor injection widths are presented in Figure 12.

In general, the evolutionary trends suggest that prey evolve to be more inclined towards gathering in smaller arenas (indicated by smaller values of $f_{g}$) and to be more prone to escaping in larger arenas (indicated by smaller values of $f_{e}$). However, as indicated by the results, the preferences of prey agents do not exhibit significant consistency across all simulation settings. This suggests that the evolution process might converge to different directions under different conditions, implying the existence of multiple fit genotypes within the given environment.

It’s worth noting that the lack of strong preference in some cases, especially for odor injection widths of $\Phi_{w}=10$ and 25, could be due to the fact that these parameter settings might not provide sufficient selective pressure, as indicated by the results shown in Figure 10 and Figure 11. However, when focusing on the results for $\Phi_{w}=30$ (represented by the blue lines in Figure 12), a clearer tendency emerges as the arena length increases: evolved prey agents become more sensitive to sensory inputs, exhibit a higher likelihood of gathering when mates are visible, and are more inclined to escape when they perceive the predator’s odor. The larger arena size offers more living space for prey agents, allowing both gathering and escaping behaviors to be preserved with higher probability.

## 4. Robot Experiments of the Prey-Predator Interaction

**Figure 13:**
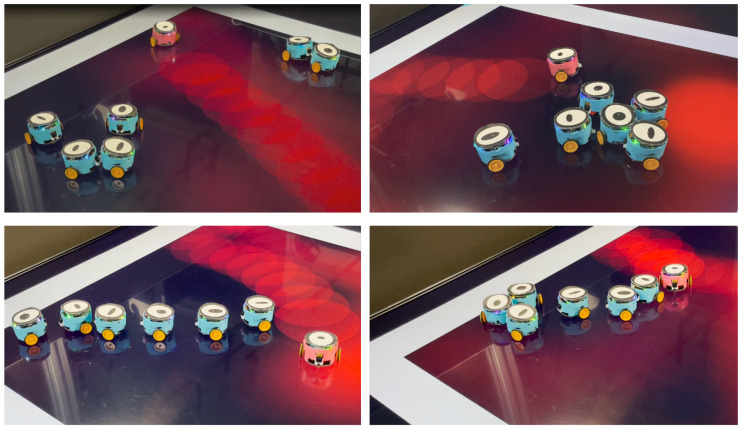
The pictures of the robots and arena captured during the experiments.

This section presents the results of experiments conducted using real robots to validate the defined prey-predator interaction system in the real world, accounting for realistic physical constraints. Four experiments were performed, each involving 6 prey robots and 1 predator robot. The experiments were conducted with different arena lengths (1200 or 800 pixels) and odor widths (80 or 100 pixels), with each experiment running for a duration of 1000 s. A brief video recording of these 4 experiments can be found in Video S2 in the supplementary material. Figure 13 displays images of the robots and the arena, featuring cyan-shelled prey and red-shelled predator robots. Prey often gather in queues or clusters, while predators roam the arena with odor fields dynamically rendered according to their real-time locations.

**Figure 14:**
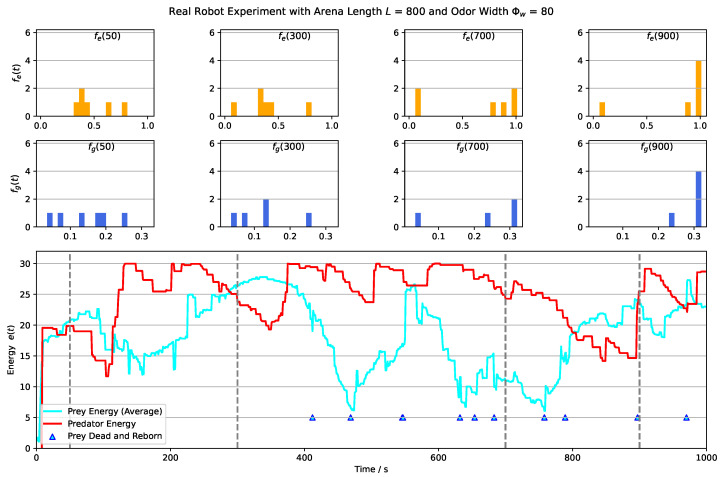
The outcomes of a real robot experiment conducted with an arena length of L=800 pixels and an odor width of $\Phi_{w}=80$ pixels. The top row of the figure showcases the distribution of the olfactory escaping factor at specific time points, while the second row illustrates the distribution of the visual gathering factor. The bottom plot provides a visualization of the energy levels of both the predator (represented by the red curve) and the prey population (represented by the cyan curve) over time. Four vertical dotted gray lines are displayed on the plots, marking the time steps that correspond to the four bar plots presented above. Additionally, triangle markers on the energy curve indicate instances when a prey robot died and subsequently reborn. These results provide insight into the dynamics of the experiment, demonstrating the interplay between the predator’s behavior, the prey population’s evolving traits, and the energy fluctuations of both groups over the course of the experiment.

Figure 14 displays the results of an example real robot experiment with an arena length of 800 pixels and an odor width of 80 pixels. The results of the other three experiments with different arena lengths and odor widths are provided in Figures S1–S3 of the supplementary materials. The video recordings of these experiments are also available in Video S2 of the supplementary materials. In the shown experiment, the prey’s evolving factors, namely the gathering and escaping factors, converge as the experiment progresses, resembling the trends observed in the computer simulations.

However, there is a notable difference in the predator’s energy levels between the real robot experiment and the simulation. In the real robot experiment, the predator’s energy cannot exceed the defined stopping threshold ($Th_{s}^{e}=30$). This discrepancy could be attributed to the number of preys present in the arena. In the real robot experiment, there are only 6 preys available as a source of energy for the predator, whereas in the simulation, there are 20 preys. This difference in the number of energy sources could explain why the predator’s energy level does not reach higher values in the real robot experiment compared to the simulation.

**Figure 15:**
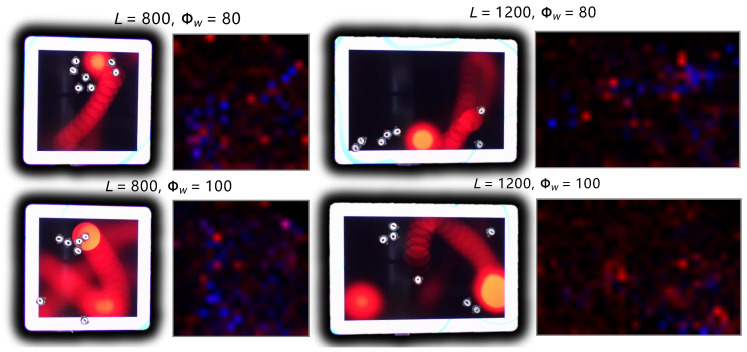
The spatial density distribution of prey and predator in four real robot experiments with different environmental conditions. The pictures with bird-eye view of the physical arena and robots are attached on the left correspondingly.

Figure 15 presents the spatial density heat maps that illustrate how the defined prey-predator interaction influences the spatial preferences of agent populations in the real robot experiment. These heat maps offer insights into the distribution and clustering of both prey and predator robots within the arena.For instance, the prey’s tendency to visually gather in queues and the predator’s dispersal of prey clusters are evident in the real robot experiments as well. These findings provide validation for the successful implementation of the prey-predator interaction scenario within the robotics platform. For a more detailed understanding, see Video S3 in the supplementary materials, which provides a visual recording of the real robot experiments.

**Figure 16:**
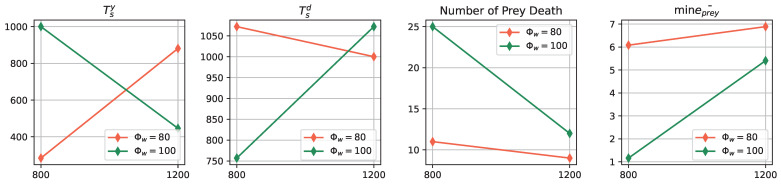
The comparison of the performances of different real robot experiments under various environmental factor settings.

To compare the results of experiments conducted under different environmental conditions, we employed several metrics: (1) $t_{s}^{y}$ and $t_{s}^{d}$ defined in (1). These metrics indicate the time steps at which the prey and predator populations reach certain energy levels, respectively, providing insights into how environmental conditions influence the energy dynamics of both populations; (2) The minimal value of the prey’s averaged energy: This metric highlights the lowest energy level reached by the prey population over the course of the experiments. It helps to understand how different environmental conditions affect the overall energy state of the prey population; (3) The number of death/rebirth events in the prey population: This metric counts the occurrences of prey robots dying and subsequently being reborn due to their energy falling below zero. It sheds light on the impact of environmental conditions on the survivability and resilience of the prey population. By analyzing these metrics across experiments conducted under different environment factors, one can deduced how variations in arena length and odor injection width influence the dynamics, energy levels, and survival of the prey population. This comparison helps us understand the sensitivity of the prey-predator interaction to different environmental settings as shown in Figure 16:

1. Impact of Odor Width: The time taken for both the prey and predator populations to reach the defined energy levels displays varying trends based on different odor widths. This suggests that the odor width has a significant impact on the energy dynamics of the populations. The inconsistent tendencies observed could arise from factors such as the Allee effect [12], which pertains to population growth being negatively impacted at low densities. In this context, with a population of only 6 preys in the arena, the sparse distribution might lead to the observed dynamics.
2. Effect of Environment on Prey Population: The number of prey deaths and the minimal prey energy levels show consistent trends across different environmental settings. Specifically, more challenging environments, such as smaller arenas with larger odor widths, lead to a greater number of prey deaths and lower minimal prey energy levels. This pattern indicates that more challenging conditions place higher selective pressure on the prey population, resulting in increased mortality and lower energy levels.

Note that the above analysis and discussion is based on the four experiments undertaken in this study, so it is hypothetical. Running more experiments could lead to more convincing conclusions. However, as this study is method-oriented, these results are sufficient to demonstrate the applicability of applying a robotics approach to studying prey-predator interactions in the real world.

## 5. Conclusions and Discussion

As the first attempt of transferring the classical prey-predator interaction study from the virtual computer world to the real world, this study has provided possible techniques to implement complex scenarios with multi-robots interacts in a multi-modal way. Implementing prey-predator interaction in the real world with physical constrains intrinsically embedded benefits the studies in many ways as that of robotics-inspired biology [2,23,29,31,38,39,40,41,42]. This study verifies the feasibility of using robotics approach to investigate the prey-predator interaction especially for that involving multi-modal sensory and evolutionary processing. In systematic simulations and several real robots experiments, the emergent spatial and temporal dynamics within the defined prey-predator have been observed and analyzed. Results suggested that emergent behaviors and embodied intelligence within the prey-predator interaction system may be unveiled through the robotics approach introduced in this study, providing a potentially useful way as a great complement of traditional researching approaches.

While the results obtained from both the simulation and real robot experiments are sufficient to validate the proof-of-concept of multimodal prey-predator interaction implemented by real robots, it is important to acknowledge that these findings represent an initial step in understanding the prey-predator dynamics. Further investigations with larger number of robots involved (especially for the real robot experiment) and systematic tuning of environmental factors are necessary to unravel the intricate mechanisms governing the dynamics of prey-predator interactions. By expanding the scale of the experiment, researchers can gain a more comprehensive understanding of emergent behaviors, population dynamics, and the role of environmental influences. Additionally, by fine-tuning other environmental factors such as the motion speed of prey and predators, the geometry shape of the arena, or the control strategy of predation, it will be possible to gain deeper insights into how these variables shape the system dynamics. Such investigations will contribute to a more robust and nuanced understanding of the intricate mechanisms driving prey-predator interactions in complex ecological systems.

While this study successfully transplanted the prey-predator interaction experiment from virtual simulations to a real robotics platform, it is important to acknowledge that there are still certain technical challenges that need to be addressed. One notable challenge lies in the wireless communication subsystem, which currently limits the number of simultaneously involved robots in the system. The communication bandwidth, range constraints and time delays impose limitations on the scalability and complexity of the experiment. To overcome this challenge, advancements in wireless communication protocols and hardware are necessary to support a larger number of robots operating in a coordinated manner. Applying other communication approach such visual coding [43] is also an option. Another challenge lies in refining the sensory capabilities of the robotic agents to accurately replicate the sensory modalities of living organisms. Although we incorporated visual and olfactory sensory cues in our system, further advancements are needed to enhance the realism and fidelity of these sensory inputs. For example, the visual input of preys is relatively simple, more objects and background scenes could be added in the future, which also make great use of the VColCOSΦ system [26]. Additionally, the locomotion and maneuverability of the robotic agents can be improved to better mimic the movements and behaviors of actual predators and prey in natural environments. Robots will different locomotion abilities like legged robot could also be incorporated in the future. Overcoming these technical issues will be crucial in advancing the application of real robotics in studying prey-predator interactions and ensuring the validity and reliability of future investigations.

## Data Availability

The data of simulation and the source codes can be found via https://github.com/XuelongSun/PreyPredatorInteractionSim (accessed on 17 September 2023).

## Supplementary Materials

The following supporting information can be downloaded at: https://www.mdpi.com/article/10.3390/biomimetics8080580/s1, Figure S1: Real Robot Experiment Arena Length = 1200 and Odor Width = 80; Figure S2: Real Robot Experiment with Arena Length = 1200 and Odor Width = 100; Figure S3: Real Robot Experiment with Arena Length = 800 and Odor Width = 100; Video S1: Simulation Animation with Arena Length = 280 and Odor Width = 20; Video S2: Real Robot Experiment Recordings; Video S3: Emergent Real Robot Behaviors.

## Appendix A. Detailed Methods

As the proposed multi-modal prey-predator interaction system is novel and has not yet been studied previously, this section presents details within the scenario for the better understanding. We hope these details along with the source code (get access via https://github.com/XuelongSun/PreyPredatorInteractionSim, accessed on 17 September 2023), could help the reader to reproduce the dynamics emergent in this study and for further investigations.

The computer simulation is implemented using Python with third-part packages *opencv* for image processing, *numpy* for matrix computation, *pyrender* and *opengl* for 3D simulated world and 2D off-screen image rendering.

### Appendix A.1. Agents

At every time step *t*, the agent’s state is denoted as: s(t)={p(t),h(t),v(t),m(t),e(t)}, where $p\left(t\right)=\left(p_{x},p_{y}\right)$ and h(t) defines its position in the arena (i.e., the *x-y* plane of the 3D simulation world) and heading respectively; v(t) is the velocity of the agent, the heading direction h(t) is always aligned with the direction of v(t) for simplicity; Therefore, agent’s moving speed along *x*- and *y*-axis can be calculated as:

(A1) $$v_{x}\left(t\right)=|v\left(t\right)|\cos h(t),\;v_{y}\left(t\right)=\left|v\left(t\right)\right|\sin h(t)$$

where |v(t)| is the speed of the agent that is determined by the controller. The position p(t) could be updated via v(t) by:

(A2) p(t+1)=p(t)+v(t)

Therefore, at every time step, the agent’s motion can be controlled simply by provides the magnitude of the velocity and the heading (or the change of the heading). m(t) is the current motion state which is depend on the agent’s internal state and environmental conditions and e(t) is the energy updated by the formula:

(A3) $$e\left(t+1\right)=e\left(t\right)+\tau \delta_{e}$$

where $\tau \delta_{e}$ is the change of the energy determined by the agent’s certain motion state and τ is the time constant.

### Appendix A.2. Environment Settings in Simulation

The simulated 3D world consists of a square-shaped arena with a side length of *L*. This arena acts as the environment in which the agents, including preys and predators, interact and exhibit behaviors. The length of the arena’s side, *L*, is considered a crucial environmental trait under investigation. Different values of *L* are used to explore the effects of this static environmental factor on population dynamics. Within the simulated world, agents are in constant motion. Preys and predators move independently based on their predefined behaviors and strategies. This dynamic movement introduces variability and complexity into the environment, influencing the spatial distribution and interaction patterns among agents. Another dynamic factor is the odor field generated by the predator’s movement. As the predator moves within the arena, it leaves behind a trail of simulated odor. The odor field dynamically evolves over time, providing a sensory cue for the preys to detect and respond to. This forms a critical component of the prey-predator interaction, where the odor acts as a stimulus for preys to exhibit specific behaviors such as escaping and gathering. The combination of static environmental traits (arena length) and dynamic factors (moving agents and evolving odor field) creates a multi-modal prey-predator interaction scenario. The agents’ behaviors are influenced by both visual cues (position of other agents) and olfactory cues (odor concentration) in their surroundings. This multi-modal interaction introduces selective pressures that drive the evolution of the agents’ behaviors over time.

The simulated 3D world provides a controlled and customizable platform for investigating the interplay between static and dynamic environmental factors and their effects on the multi-modal prey-predator interaction. This setup allows researchers to gain insights into the underlying mechanisms driving emergent behaviors in swarm robotics scenarios.

#### Appendix A.2.1. Population Energy

We consider energy as the primary variable for assessing the performance of each agent population. As previously explained, preys expend energy during activities like escaping and wandering, while they replenish energy when stationary within a cluster formed by gathering with their peers. On the other hand, the predator expends energy for odor release and gains energy from preys that overlap spatially with the odor field. This overlap is computed using the following formula:

(A4) $$e_{p}\left(t\right)=\alpha \sum_{i=1}^{N_{prey}^{a}}e_{i}\left(t\right)$$

where $N_{prey}^{a}$ is the number of preys whose motion state is ‘escaping’, namely, $N_{prey}^{a}=\#\left\{i|m_{i}\left(t\right)=2\right\}$; α is the scale factor and is set to be 0.008 in the simulation.

#### Appendix A.2.2. Predator’s Odor Field

The odor field Φ(t)=Φ(x,y,t) released by the predator (see examples in Figure 3) is updated by:

(A5) $$\frac{\partial \Phi (x,y,t)}{\partial t}=\frac{\partial \Phi^{I}\left(x,y,t\right)}{\partial t}+\frac{\partial \Phi^{D}\left(x,y,t\right)}{\partial t}+\frac{\partial \Phi^{E}\left(x,y,t\right)}{\partial t}$$

which represents three significant characteristics of odor:

(1) injection ($\Phi^{I}$): predator will release the odor at its position:

(A6) $$\frac{\partial \Phi^{I}\left(x,y,t\right)}{\partial t}=k^{I}\delta (x-p_{x},y-p_{y})$$

where $\left(p_{x},p_{y}\right)$ is the current position of the predator, $k^{I}$ is a constant number scaling the strength of the injection of odor; In the discrete format:

(A7) $$\frac{\Delta \Phi^{I}\left(x,y,t\right)}{\Delta t}=k^{I}\;if\;|x-p_{x}|\leq \Phi_{w}\;\&\;\left|y-p_{y}\right|\leq \Phi_{w}$$

(2) diffusion ($\Phi^{D}$): the pheromone will spread along *x* and *y* direction in the 2D plane, which can be described by the *diffusion equation*:

(A8) $$\begin{array}{c} \frac{\partial \Phi^{D}\left(x,y,t\right)}{\partial t}=k^{D}\nabla^{2}\Phi \left(x,y,t\right) \end{array}$$

In the discrete format, this operation could be implemented by convolution:

(A9) $$\Delta \Phi^{D}\left(t\right)={k^{D}}^{\prime }\Phi \left(t\right)*G^{D}\Delta t$$

where $G^{D}$ is the kernel:

(A10) $$G^{D}=\left[\begin{array}{ccc} 1/8 & 1/8 & 1/8\\ 1/8 & -1 & 1/8\\ 1/8 & 1/8 & 1/8 \end{array}\right]$$

and ${k^{D}}^{\prime }$ is a constant determines the contribution of the diffusion to the odor change. Note that in the discrete implementation given the time interval Δt, the overall impact of diffusion factor $k^{D}$ is separated into two parts: the size of the kernel (3×3) and the constant ${k^{D}}^{\prime }$.

(3) evaporation ($\Phi^{E}$): the concentration of the odor at location (x,y) will decrease exponentially with respect to time.

(A11) $$\frac{\partial \Phi^{E}\left(x,y,t\right)}{\partial t}=-k^{E}\Phi \left(x,y,t\right)=-\frac{1}{\tau^{E}}\Phi \left(x,y,t\right)$$

where $k^{E}$ is a constant defines the time constant ($\tau^{E}$) of exponential decay. In the discrete time format:

(A12) $$\frac{\Delta \Phi^{E}\left(x,y,t\right)}{\Delta t}=-\frac{1}{\tau^{E}}\Phi \left(x,y,t-1\right)$$

#### Appendix A.2.3. Prey’s Evolution

Within the prey population, the energy variable e(t) plays a critical role in determining the survival of individual preys. For instance, if the energy level falls below zero (e(t)<0), the prey will perish, prompting the generation of a new prey to sustain the evolutionary process. This process is influenced by two genotypes, namely $f_{e}$ and $f_{g}$ as depicted in Figure 2C. These genotypes govern the escaping and gathering behaviors of the prey, and they are inherited by the new spawns from the existing prey with the highest energy levels. In order to diversify and prevent the evolutionary process from converging to local extrema, an element of randomness is introduced. Specifically, a 10 percent probability is assigned for randomly selecting an existing prey. Subsequently, the newly generated prey will inherit the values of $f_{e}$ and $f_{g}$ from the chosen prey, fostering greater exploration of the genotype space.

#### Appendix A.2.4. Parameters

Table A1 lists the main parameters that were utilized throughout all the simulations. Notably, the number of agents in each group remained constant (20 preys and 1 predator). Instances where values are highlighted in red in Table A1 signify that these values were individually selected to conduct systematic experiments. This approach, while diverging from conventional studies where population size is a key variable, allows us to utilize energy as a corresponding factor to investigate population dynamics. The specific values for the arena length *L* and the odor injection width $\Phi_{w}$ were systematically adjusted according to the red text in Table A1. For each parameter configuration, we conducted 10 trials, resulting in a total of 4×5×10=200 trials. Each simulation trial was executed for a duration of T=6000 steps.

**Table A1:** The parameters setting in simulations. Red text indicates that these values of the certain parameter have been used to undertake the evaluating experiments.

Category	Parameter	Value	Description
Environment	L	200, 240, 280, 320	environmental factor
Predator	$\Phi_{w}$ (Figure 3)	10, 15, 20, 25, 30	prey-predator interaction factor
	$k^{I}$ (Equation (A7))	100	odor field
	$k^{D^{\prime }}$(Equation (A9))	0.99	
	$k^{E}$ (Equation (A11))	1000	
	$N_{predator}$	1	the number of preys is constant
	$Th_{s}^{e}$ (Figure 3)	100	threshold of energy for stop hunting
	e(0)	50	initial energy of predator population
	|v(t)|	8	
Prey	$N_{prey}$	20	the number of preys is constant
	e(0)	2	initial energy of prey population
	$f_{g}$	U(0.02,0.32)	initial value is randomly generated
	$f_{e}$	U(0.01,10.01)	
	$Th_{s}$ Figure 2	0.4	threshold (percent of image pixels) for stop gathering
