# Supplementary figures and images for: Translating Virtual Prey-Predator Interaction to Real-World Robotic Environments: Enabling Multimodal Sensing and Evolutionary Dynamics
Source: Biomimetics (Basel). 2023 Dec 1;8(8):580. doi: 10.3390/biomimetics8080580 (PMC10742093; doi:10.3390/biomimetics8080580)

Real Robot Experiment with Arena Length  $L = 1200$  and Odor Width  $\Phi_w = 80$

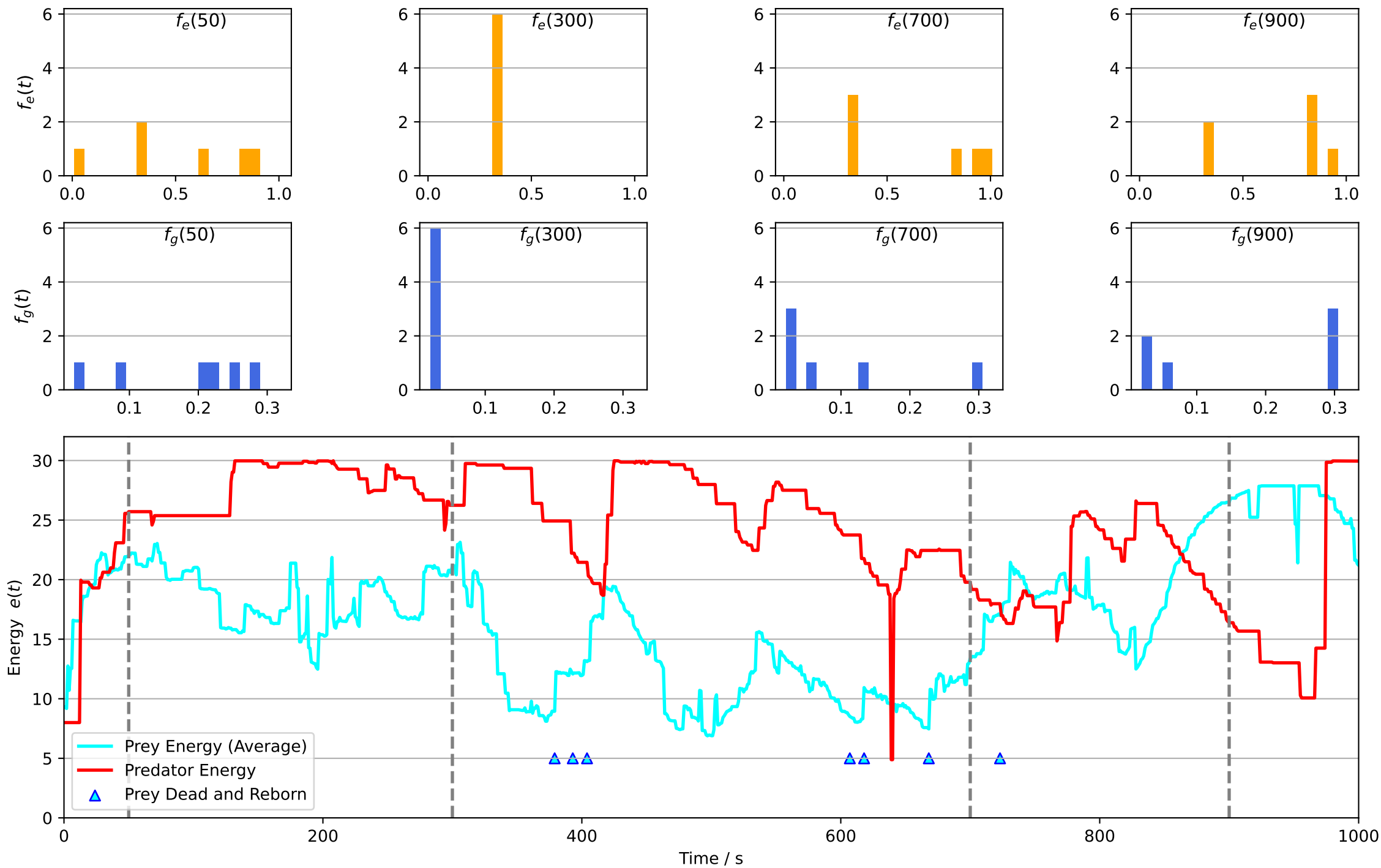

Supplement: Supplementary file 1 [file biomimetics-08-00580-s001.zip › FigS1-RealRobotExperimentArenaLength1200OdorWidth80.pdf]

Real Robot Experiment with Arena Length  $L = 1200$  and Odor Width  $\Phi_w = 100$

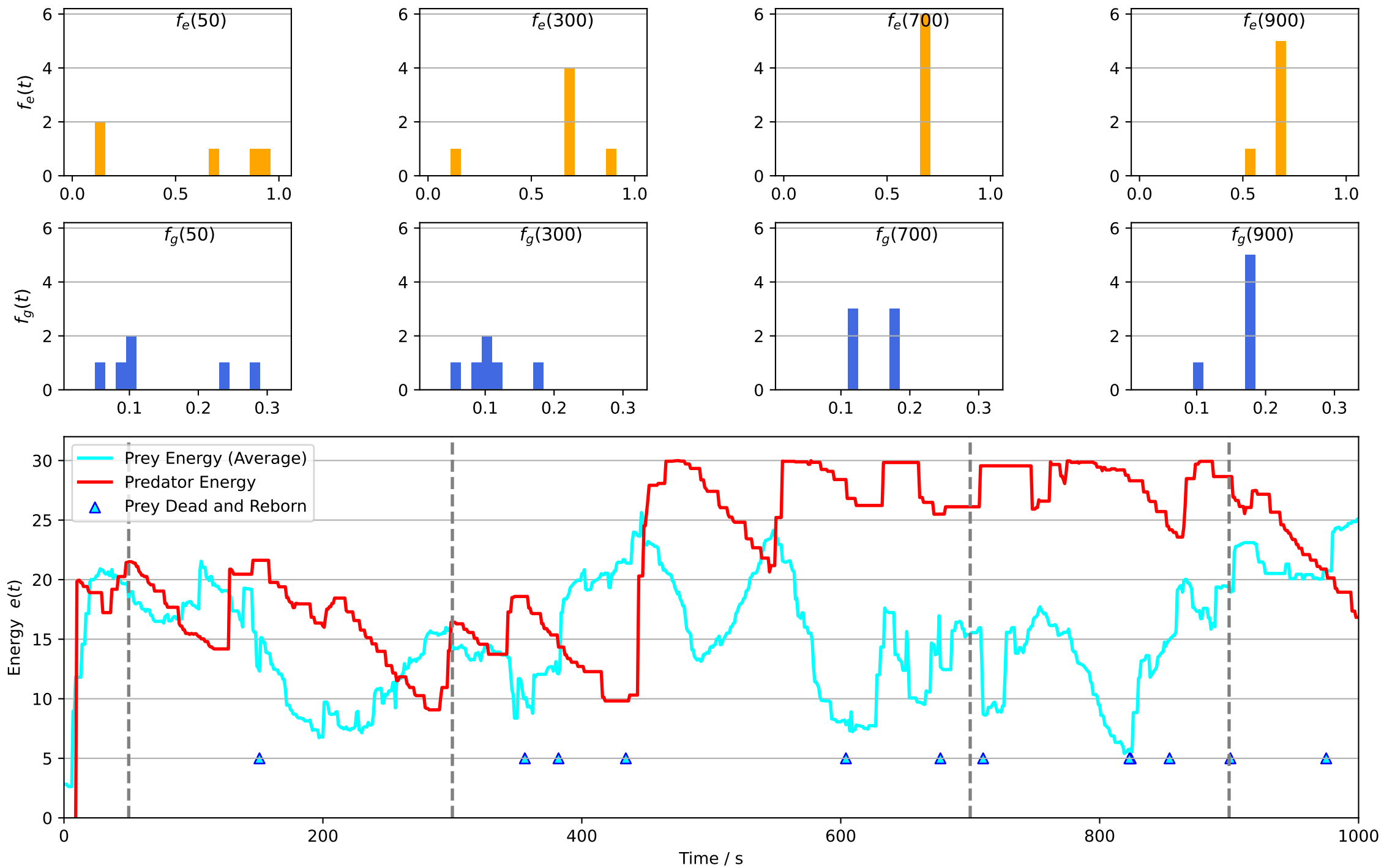

Supplement: Supplementary file 1 [file biomimetics-08-00580-s001.zip › FigS2-RealRobotExperimentArenaLength1200OdorWidth100.pdf]

Real Robot Experiment with Arena Length  $L = 800$  and Odor Width  $\Phi_w = 100$

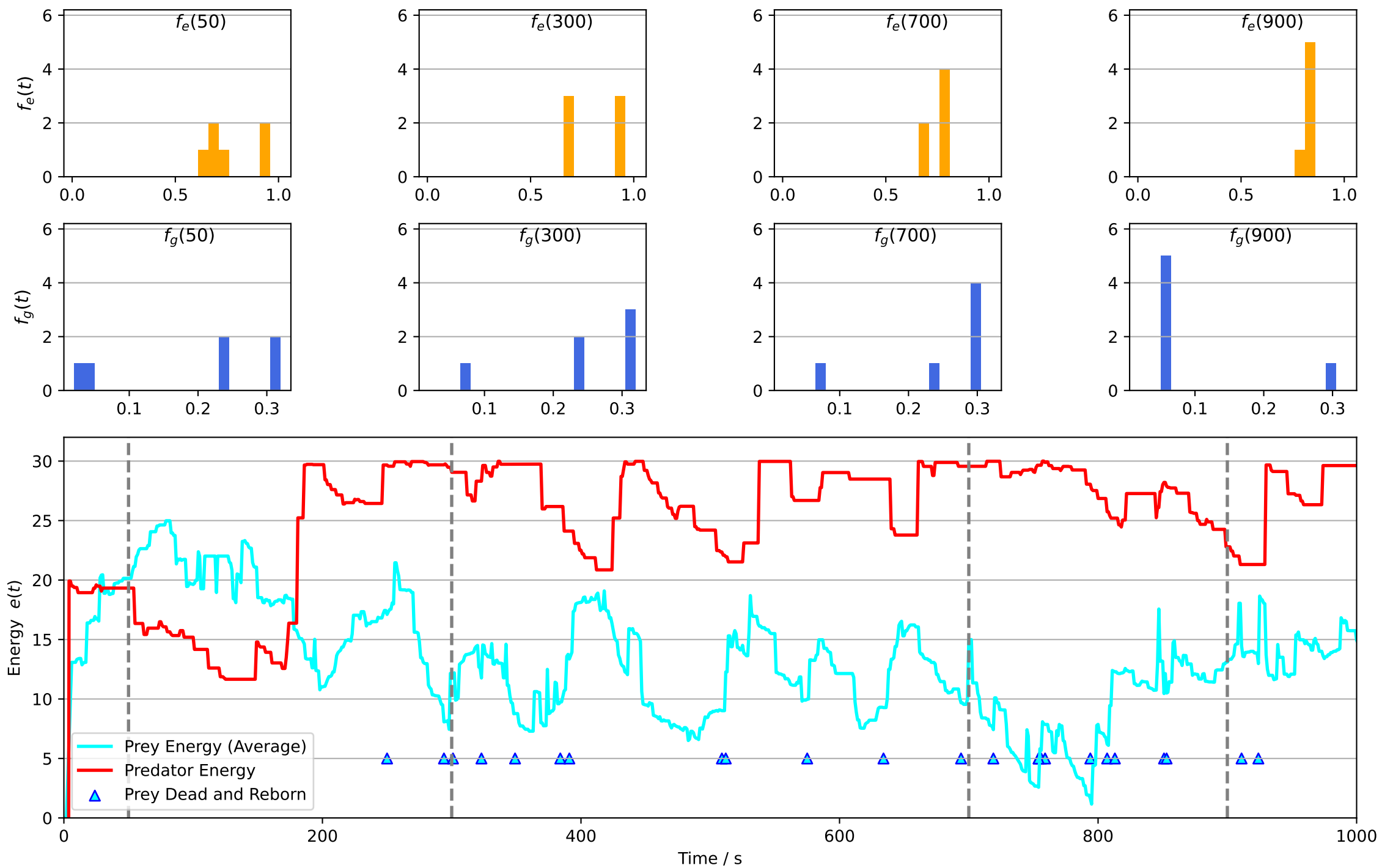

Supplement: Supplementary file 1 [file biomimetics-08-00580-s001.zip › FigS3-RealRobotExperimentArenaLength800OdorWidth100.pdf]
